# Molecular and modular intricacies of precision oncology

**DOI:** 10.3389/fimmu.2024.1476494

**Published:** 2024-10-23

**Authors:** Ravneet Chhabra

**Affiliations:** Business Department, Biocytogen Boston Corporation, Waltham, MA, United States

**Keywords:** precision oncology, molecular biomarkers, artificial intelligence, clinical trials, personalized treatment

## Abstract

Precision medicine is revolutionizing the world in combating different disease modalities, including cancer. The concept of personalized treatments is not new, but modeling it into a reality has faced various limitations. The last decade has seen significant improvements in incorporating several novel tools, scientific innovations and governmental support in precision oncology. However, the socio-economic factors and risk-benefit analyses are important considerations. This mini review includes a summary of some commendable milestones, which are not just a series of successes, but also a cautious outlook to the challenges and practical implications of the advancing techno-medical era.

## 1 Introduction

Oncology therapies are commonly designed to target the highly dysregulated molecular pathways, including Ras/MAPK, Myc, Wnt/β-catenin, TGFβ, PI3K/mTOR, Notch signaling, Hippo pathway, cell cycle, oxidative stress response and/or p53 signaling (1–3). However, therapeutic resistance poses a constant struggle, whether it is ‘intrinsic’ due to genetic/molecular dysregulations or ‘acquired’ due to cancer cells adapting to the cellular changes (4). Tumor heterogeneity, complex tumor microenvironment and genetic predisposition have complicated the treatment options further. Personalized treatment approaches are therefore successfully proving to be the present and future of medicine. Precision Oncology is constantly evolving to acknowledge, accept and utilize every human being’s uniqueness, characterized by a distinct set of genetic make-up (5, 6). As the “one size fits all” theory is challenged at various levels in therapeutic arena, precision medicine has emerged to rescue the unique individual cases (7–9), where common FDA approved chemotherapeutics and/or immunotherapy drugs fail to eliminate the cancer cells (10–13).

As with great power comes bigger economic impact, personalized healthcare requires large sums of investments and some of the underrepresented or minority groups may have limited access to such novel technologies (14). This coincides with Eroom’s law, which describes the ever-slowing rate of drug discovery and applicability with increasing costs associated with it (15). This further widens the gap between research and its practical applications (15). Balancing the resources with medical goals, patient requirements, time involved, and risk assessment is critical.

Although there are multiple tools used to support the personalized approach, attempting to reverse the Eroom’s law, one of the approaches gaining traction is incorporation of artificial intelligence/machine learning (AI/ML) into biotechnological advancements (16). Since 2016, FDA has seen an exponential increase in usage of AI/ML to new oncology clinical trials, in different phases from patient recruitment and precise clinical designs (17) using de-identified electronic health records (18) to data collection and analysis (19–22). These technologies provide a major boost to generating customized treatment plans for specific groups/sub-groups/individuals based on the target mutations. AI/ML algorithms can identify complex patterns and correlations by analyzing large datasets, which may not be possible at human/physician level (22).

Targeting the molecular and cellular characteristics of tumors has been the focus of precision medicine for decades (23). Genetic profiling methods combined with immunophenotyping, transcriptomics and epigenetic analyses assist in de-coding the complex deregulated pathways of tumor microenvironment at a high throughput level, while conventional methods such as FISH (Fluorescence *in situ* hybridization) and IHC (Immunohistochemistry) are commonly used to detect predictive biomarkers (24). Some common immunotherapeutic drugs targeting PD-1/PD-L1 (nivolumab), CTLA (ipilimumab), TIGIT (tiragolumab), LAG3 (Relatlimab) are well-studied and used by clinicians (25–27). However, in cases concerning rare cancers (such as angiosarcoma (28), metaplastic breast cancer (29)), high risk, relapsed or refractory pediatric cancers (such as Neuroblastoma (30), pediatric brain tumor (31), medulloblastoma, Wilms’ tumor (32, 33)), and resistant cancer sub-types (characterized by overexpression of HER2, Ras/MAPK pathways (34)), customized/personalized cell therapy, gene therapy, immunotherapy, and/or a combination of treatments in a timely manner can successfully aim to prolong symptom free survival in cancer patients (35–41).

## 2 Current landscape of precision oncology therapy

**Figure 1:**
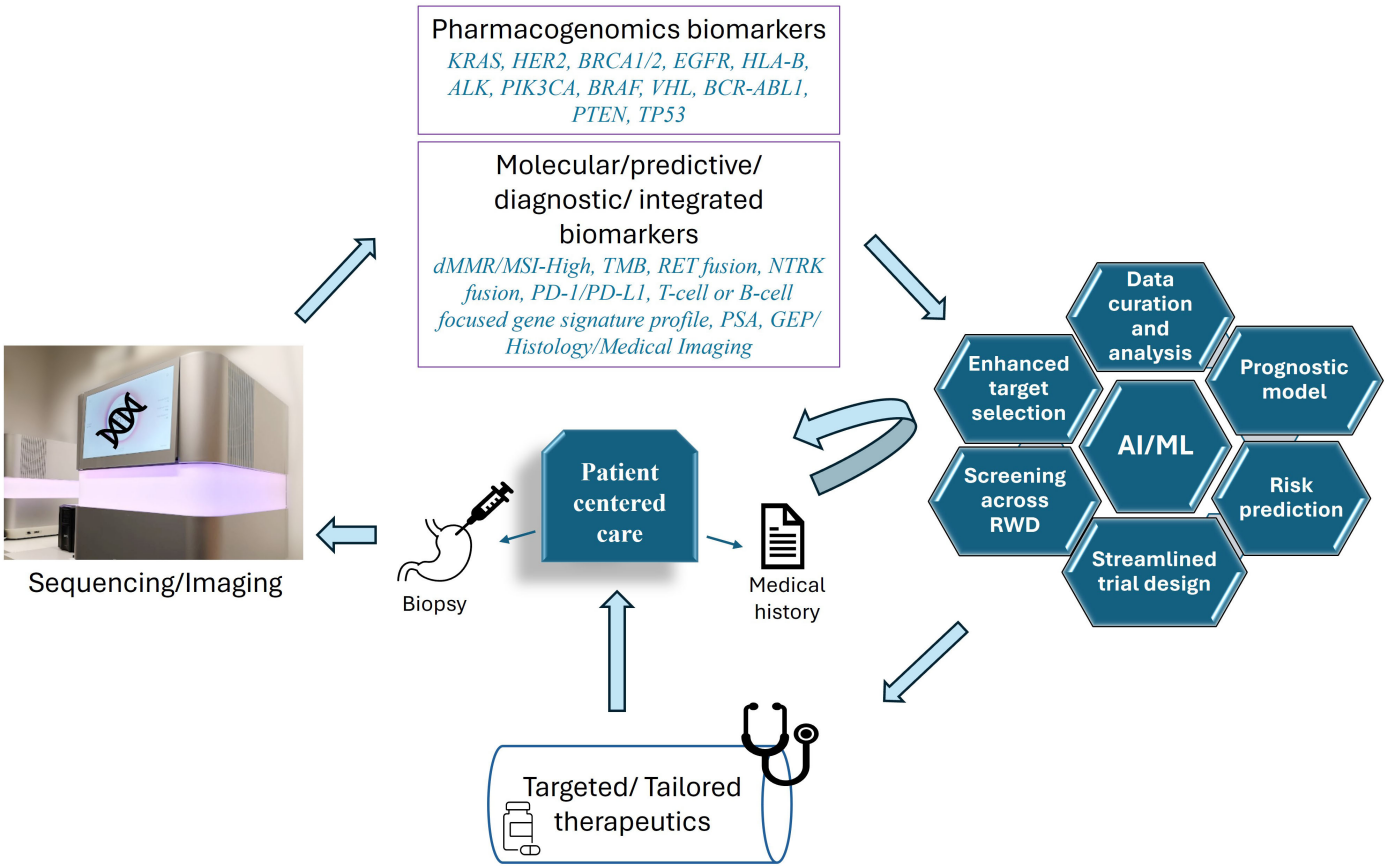
Patient centered personalized care for cancer treatment. To understand the unique molecular make-up of each patient, biopsy samples are analyzed via different sequencing techniques, (including genetic, epigenetic, RNA and ncRNA sequencing) and through medical imaging/histological analysis. These techniques potentially reveal the different pharmacogenomic markers commonly altered in different cancer types, including but not limited to, KRAS (Kristen Rat Sarcoma Viral oncogene homolog), HER2 (human epidermal growth factor receptor 2), BRCA1/2 (breast cancer gene), EGFR (epidermal growth factor receptor), HLA-B (human leukocyte antigen B), ALK (anaplastic lymphoma kinase), PIK3CA (Phosphatidylinositol-4,5-Bisphosphate 3-Kinase Catalytic Subunit Alpha), B-RAF (v-raf murine sarcoma viral oncogene homolog B1), VHL (Von Hippel-Lindau), BCR-ABL1 (breakpoint cluster region and Abelson murine leukemia viral oncogene homolog 1), PTEN (phosphatase and tensin homolog deleted on chromosome 10), TP53 (tumor protein p53) (7, 8, 168). Additionally, different molecular patterns, biomarkers or integrated panels are also analyzed, serving as predictive or diagnostic signatures. These include, but are not limited to, dMMR (deficient DNA mismatch repair)/MSI-high (microsatellite instability-high), TMB (Tumor mutation burden), RET (REarranged during Transfection) genetic fusion, NTRK (neurotrophic tyrosine receptor kinase gene) fusion, PD-1/PD-L1 (Programmed Cell Death Protein 1 and Programmed Cell Death Ligand 1), T-cell or B-cell focused gene signature profile, PSA (prostate-specific antigen), GEP (T cell–inflamed gene expression profile), tumor imaging and histology (34). AI/ML is used for a myriad of functions such as sorting the markers, screening across real world data (RWD), generating prognostic models, risk prediction, selecting specific targets, and testing drug combinations (169). Patient-derived data (based on tested samples and medical history) is curated and streamlined not only for specific clinical trial design, but also to record, statistically analyze, and compare the results (169).

Modern clinical medicine relies on the 4Ps, serving as pillars to support therapeutic decision making, namely, Predictive, Preventative, Personalized and Participative approach, focusing on robust treatment options in a patient-centric framework (42). Treating the patients with the right medicine at the right time is always the clinical goal, however, the concept of “personalized treatment” has evolved within the last few years. The success and FDA approval of HER2-specific breast cancer targeting drug: trastuzumab in 1998 (43, 44) and BCR-ABL tyrosine kinase inhibitor Chronic Myeloid leukemia drug: Imatinib in 2001 (45) were the first major stepping stones in this field, followed by a wide range of gene-targeting treatment options (46). As cancer is described as both genetic and molecular group of diseases, it became important to encompass other intricate alterations involved, such as epigenetic factors (47), biomarkers (48) and anatomical/histological modifications (49) to understand the disease progression and design individualized “precision medicine” treatments (**Figure 1**).

Gene and molecular-targeted therapy (designed to target only cancer cells) and Immunotherapy (used to boost body’s immune system against cancer), are the major approaches used individually or in combination with chemotherapy and/or radiotherapy to treat cancer patients. Within last 20 years, a plethora of drugs, including checkpoint inhibitors, monoclonal/bispecific antibodies, antibody-drug conjugates, chimeric antigen receptor T (CAR-T) cells have been developed to combat the complexities of this disease. The approval of blinatumomab, the first bispecific antibody in 2014 (50) and tisagenlecleucel, the first CAR-T cell therapy in 2017 (51) marked milestones in oncology research. Based on OncoKB (RRID : SCR_014782) (updated June 19, 2024), FDA has approved 186 new targeted therapy drugs since June 1998, out of which 96 are precision oncology drugs.

CAR-T therapy is a highly promising treatment for hematological malignancies. As in most cases it works by using patients’ own T-cells (autologous), this therapy is highly precise and effective (52). Peripheral blood derived T cells are genetically modified to integrate CAR expression cassette into the genome, and CAR proteins are subsequently expressed on surface of T-cells. These modified cells are expanded and infused back into the patients. CAR recognizes specific cancer antigens, forms an immune synapse and lyses the tumor cell by activating granzyme-perforin axis, Fas/Fas ligand pathway and release of cytokines (52). So far, six CAR-T therapies have been FDA approved for use in clinics, targeting two antigens- CD19 and BCMA (53). However, owing to the long term adverse effects of CAR-T, such as cytokine release syndrome and neurological toxicity (54), further research and advancements are moving this field forward, such as integration of CAR with other immune cells - NK/NKT cells, dendritic cells, macrophages, regulatory T cells and B cells (55, 56), which may have the potential to be safer for long-term use and hold high therapeutic potential for clinical use.

Moreover, metabolic dysregulation is a well-known phenomenon in tumors, characterized by accelerated glycolysis, upregulation of lipid and amino acid metabolism, alterations in mitochondrial biogenesis and macromolecule biosynthesis- all of which are considered hallmarks of cancer (57). Various chemotherapeutics targeting the altered molecules in metabolic machinery are well established for clinical use. Some examples include enasidenib for mutated isocitrate dehydrogenase 2 (IDH2) and Ivosidenib for mutated isocitrate dehydrogenase 1 (IDH1) in acute myeloid leukaemia (AML), 5-fluorouracil inhibiting Thymidylate synthase in gastric and breast cancer, and Methotrexate inhibiting dihydrofolate reductase (DHFR) in breast and lung cancer (58, 59). However, activation of DNA repair pathway, induced apoptosis resistance, target alterations, and reprogramming of immune cells by limiting nutrient availability within tumor microenvironment can lead to resistance towards these therapies (60, 61). Understanding the overall picture of the tumor complexity reinforces the concept of combination therapy precisely designed to target the cancer cells from various angles (58, 62).

Traditionally, clinical trials are drug-centered, blinded and randomized to minimize bias. However, due to large variability in patients’ tumor microenvironment, molecular profiles and unique genomic characteristics, the outcomes are far from ideal (63). Therefore, innovative patient- centered trials are now customized to recognize genomic alterations and employ novel biomarker-guided methodologies to address the distinctive needs of patients (64) (**Figure 1**). A unifying clinical trial framework known as master protocols includes testing multiple drugs in parallel, for patients with same or different types of cancer (65). The major trial designs under master protocols are summarized in **Table 1** and are detailed as follows:

**Table 1:** This table summarizes the precision oncology clinical trial types, unified as master protocols.

Trial Type	Examples	Purpose	Challenges
Basket	NCI-MATCH, NCI-MPACT, TAPUR (70–72)	Tests new drug in multiple cancer types with pan-cancer gene defect or biomarkers	Tumor heterogeneity leading to less accurate prediction of response rates; Lack of appropriate controls (73)
Umbrella	Lung Matrix, myeloMATCH, ALCHEMIST, I-SPY-2, plasmaMATCH (74–79)	Tests multiple therapies in one disease group with common histological aberration (sub-grouped with different biomarkers or genomic sub-sets)	Slow enrollment process due to sub-grouping of patients (80)
Platform	ComboMATCH, SHIVA (81, 82)	Tests multiple drugs against a common disease with flexibility of modifications, as needed	High cost and time duration due to higher complexity (80)
Octopus	QUILT-3.055 N-803 in patients who received pre-treatment with PD-1/PD-L1 immune checkpoint inhibitors (83)	Tests the combinatorial effects of multiple drugs by simultaneously investigating several treatment arms	Interdependent data generation may lead to statistical limitations (84); high cost
N-of-1	I-PREDICT, rare pediatric cancer cases (86–89)	Matches patients to drugs and RWD, effective for rare, resistant and metastatic cancers	Suboptimal controls; conservative data collection and analysis; statistical limitations; false-negatives and high cost (83)

### 2.1 Basket trials

These include testing of a new drug in patients with common genetic mutation (pan-cancer gene defect) or biomarkers, in more than one cancer types (66). Common examples of drugs targeted to specific genes include Pembrolizumab for tumor mutational burden high (TMB-H) and mismatch repair deficiency/microsatellite instability high (dMMR/MSI-High) (67), and Larotrectinib (68) and entrectinib (69) in tumors with NTRK fusion. Well known basket trials such as NCI‐MATCH (Molecular Analysis for Therapy Choice) (70) and National Cancer Institute’s Molecular Profiling‐Based Assignment of Cancer Therapy (NCI-MPACT) (71), TAPUR (Targeted Agent and Profiling Utilization Registry) (72) were phase 2 trials based on molecular profiling of different cancer sub-types. In some cases, such trials may not accurately predict the response rates due to heterogeneity of the tumors and appropriate control groups may not be available (73).

### 2.2 Umbrella trials

Test of multiple therapies in one disease group with common histological aberration, stratified in sub-groups based on different biomarker or genomic subsets. Some examples include The Lung Matrix trial (74), Myeloid Malignancies Molecular Analysis for Therapy Choice (myeloMATCH) (75), Adjuvant Lung Cancer Enrichment Marker Identification and Sequencing Trial (ALCHEMIST) (76, 77), Investigation of Serial Studies to Predict Your Therapeutic Response With Imaging And moLecular Analysis 2 (I-SPY-2) (78), and The UK plasma based Molecular profiling of Advanced breast cancer to inform Therapeutic Choices (plasmaMATCH) (79). One challenge with these trials is the requirement of sub-grouping of patients that could slow down the enrollment process in case of rare cancers (80).

### 2.3 Platform trials

They are also known as multi-arm, multi-stage design trials which include testing multiple drugs against a common disease. Based on the interim analysis, these trials allow changes to the ongoing trial *vis à vis* addition of a control arm, drug, patient population or even early termination, as needed (80). This flexibility enables platform trials to be confirmatory. Some examples include ComboMATCH (NCI Combination Therapy Platform Trial with Molecular Analysis for Therapy Choice) (81), and SHIVA (Study of Randomized, Molecularly Targeted Therapy Based on Tumor Molecular Profiling versus Conventional Therapy for Advanced Cancer) (82). Since platform trials are large scale and logistically complex, the cost and time duration involved could be high (80).

### 2.4 Octopus trials

These are completed Phase I/II trials, which evaluate the combinatorial effects of multiple drugs with a common intervention (83). An example is phase IIb multi-cohort study QUILT-3.055, which tests combinations of N-803 (a fusion protein inducing proliferation and activity of natural killer and cytotoxic T-cells) in patients who received pre-treatment with PD-1/PD-L1 immune checkpoint inhibitors (83). Since these trials are multi-arm, data generation could be interdependent, leading to potential statistical limitations (84).

### 2.5 N-of-1 trial

Randomized and blinded trial conducted in a single patient. These are, in a true sense, personalized trials based on specific biologic characteristics (85). These can be effective in treating rare cancers and to provide objective comparison of different treatments and perform time series analyses (83). Various N-of-1 trials are comprehensively summarized by Gouda et al. (86). Some examples include I-PREDICT study (87), rare pediatric cancer, such as- the case of a 2-year old child with metastatic glomus tumor and activated NOTCH1 (88), and the ALK-fusion positive high grade glioma in a 3-year old (89). However, there are serious considerations to performing these trials, ranging from lack of appropriate control and highly conservative treatment selection to data collection and analysis, statistical limitations, false-negatives and the high cost involved in putting together the infrastructure for each trial (83).

Since the patient-centric biomarker-based studies rely on appropriate detection of the relevant disease indicators, several methods are used to analyze and aid in designing the treatment plans.

## 3 Onco-precision toolkit

Technological advancements in cancer biology have enabled researchers and clinicians to explore options beyond the common drug targets for patients. Even though the DNA sequencing techniques have been in use since 1970s (90), the most widely accepted next generation sequencing (NGS) was adapted in clinical diagnosis and prognosis within the last decade (91). With the development of clinical applicability of whole genome sequencing (92, 93), whole exome sequencing (WES) (94, 95), RNA-seq (paired with WES (96)) or in single-cell/bulk variations (97)), spatial transcriptomics (98), hybrid capture NGS for targeted oncology panels (99) and comprehensive omics analyses (100, 101), the integration of large-scale genomic data (102) is now possible to drive the personalized treatment approaches. Besides the genetic mutations at DNA and RNA level, several ncRNAs such as miRNA (103, 104), circular RNA (105–107) as well as epigenetic markers (108–111) are being analyzed to comprehensively map individuals at genetic and molecular level. These techniques draw the cellular landscape of tumors and help in discovering biomarkers associated with clinically relevant genomic alterations, as summarized in **Figure 1**.

Radiomics or high dimensional medical imaging via PET, CT, Ultrasound and MRI (112) for monitoring the tumor characteristics is combined with machine learning to extract the specific features/characteristics of individual tumors, to guide their specific treatment course (113, 114). Theranostics (therapy + diagnostics) utilizes radionuclide linked to targeted biomarkers, which allows diagnosis through targeted imaging (radiomics) and targeted therapy at the same time (115). Some examples include Lutathera (lutetium ^177^Lu dotatate), the first FDA and EMA approved theranostic drug, which releases radiation to kill cancer cells by binding to cell surface receptors somatostatin on gastroenteropancreatic neuroendocrine tumors (116), and Pluvicto for castration-resistance prostate cancer using Lutetium-177 that targets PSMA on cell surface in prostate cancer (117).

**Figure 2:**
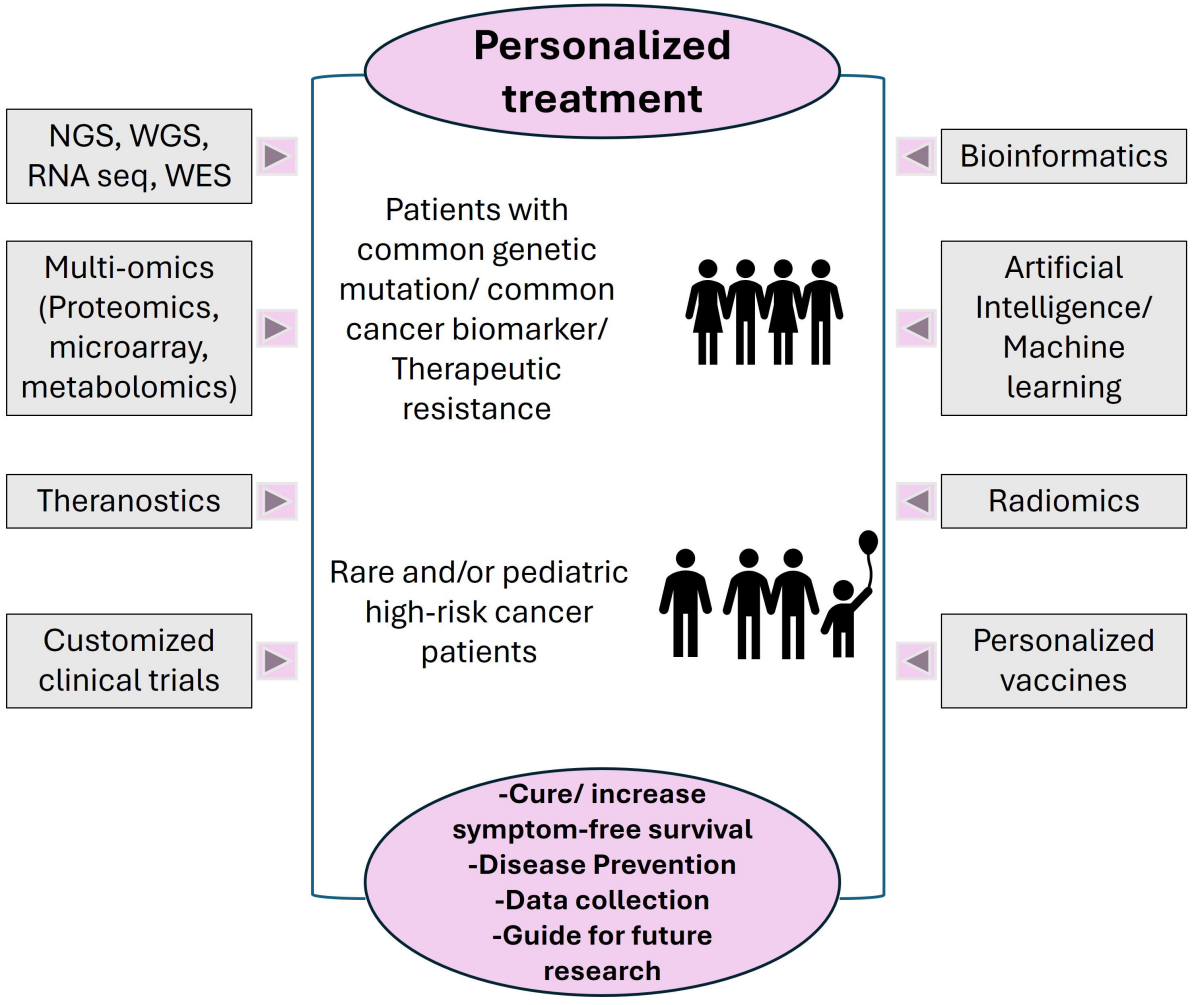
Different Precision Oncology tools utilized for diagnosis, prognosis, data collection and analysis. The target population for personalized treatment includes, but is not limited to, patients that share genetic dysregulation/common biomarkers altered/patients with therapeutic resistance, patients with rare cancer types, pediatric patients with high-risk, relapsed or refractory cancers. The current goals are defined by achievable level of cure or longevity of symptom free survival; some tools such as NGS, preventative personalized vaccines and specific AI/ML technologies can be used for prevention or early precautionary measures. The data collected can also be stored in repositories to guide treatments for other patients, can be used as training data for next generation of advanced technologies and to design novel future medical interventions.

Various ML methods, such as deep neural networks are also used to predict clinical outcomes using the supervised and unsupervised learning models (118, 119) to enhance the efficiency of cancer diagnosis and increase the probability for predictive prognosis. With the use of de-identified electronic health records (EHRs) (120), paired with specific genotypic training data (121) and bioinformatic regression models (122), the auto-encoders can extract intrinsic features of the tumors (118). This high throughput real-world data (RWD) paves the way to deeper understanding of complex biomarkers associated with heterogenous tumor sub-populations, microsatellite instability and tumor mutational burden (TMB) (123–125). **Figure 2** summarizes the tools available for personalized cancer treatment for specific population groups, to achieve the clinical goals of treatment and prolonged survival.

Various governmental organizations such as NCI, NCBI and FDA provide open access public repositories to study the pharmacogenomic pattern of larger population groups with drug response, increased clinical efficacy probability and reduced adverse drug reactions. Some examples include TCGA (reports molecular characterization of 20,000 primary tumors) (RRID : SCR_003193), ClinVar (public archive of human diseases and corresponding drug responses) (RRID : SCR_006169), COSMIC (catalogue of somatic mutations in cancer) (RRID : SCR_002260), PharmKGB (Pharmacogenomics Knowledgebase for genotype-phenotype relationship, genetic variants and drug associated guidelines) (RRID : SCR_025580), Drugbank (for comprehensive drug-target data) (RRID : SCR_002700), FDA’s pharmacogenetic associations and ClinGen (human genetic variants database) (RRID : SCR_014968) (9, 126). Precision FDA is also a free computing platform to analyze large biological datasets and learn from experts in the field.

Utilization of these vast array of tools available carries its fair share of challenges ranging from the cost and time involved in generating the large datasets to managing, storing, aligning and assessing this data, with high quality, accuracy and reproducibility. Aligning multi-reads, incorrect sequence mapping, absence of reference sequences, computational challenges spanning splice or fusion junctions, misalignment and false-positive identification are a few common problems noted with NGS and RNA-seq methods (127, 128). At experimental level, the quality of RNA and DNA extracted from formalin fixed embedded (FFPE) tissues derived from tissue banks may not be the best in some cases for high throughput analyses (129).

Moreover, clonal diversity and tumor heterogeneity is a major challenge in a constantly evolving tumor microenvironment, which can interfere with accurate detection of driver mutations and novel factors leading to resistance towards therapy. Common examples of acquired resistance include splice variants affecting ATP-competitive tyrosine kinase inhibitor binding sites, activating or sub-clonal mutations in PI3K, RAS/MAPK pathways, mutations in “undruggable” genes such as Myc, KRAS and Tp53, FLT3 mutant leukemia (130), and somatic mutations in cancer stem cells (4). Realistically, biopsy at recurrence or relapse is not always possible in case of severe metastasis associated with procedure invasiveness and underlying co-morbidity (131, 132). However, using resources like NCI-MATCH and pairing them with sequential screening tests of samples derived from liquid biopsies, and circulating tumor (ct)-DNA based targeted sequencing (133) based on specific genetic panels can be a way to detect actionable genomic alterations and predict the resistance to adapt customized approaches.

## 4 Cancer vaccines: a long journey with promising outcome

Cancer vaccines can be categorized into (a) Preventative, such as hepatitis B vaccine and human papilloma virus (HPV) vaccine, administered to reduce the risk of liver cancer and cervical cancer, respectively (134), or (b) Therapeutic, such as Sipuleucel-T against metastatic prostate cancer (134), Nadofaragene firadonevec (Adstiladrin) for early-stage bladder cancer (135), and T-VEC (Imlygic) to treat advanced melanoma (136).

Although the first cancer vaccine trial dates back to 1890s, when William B. Coley used heat-killed streptococcal injections in patients with inoperable sarcomas (137), a major leap forward was in 1959 when Llyod Old showed that BCG infection in mice increased their resistance towards transplanted murine tumor cell lines S-180, carcinoma 755 and Ehlrich ascites (138). The BCG vaccine containing live attenuated Mycobacterium bovis was later approved by FDA for early-stage bladder cancer (139).

As preventative cancer vaccines have limited applicability and efficacy against the plethora of cancer-causing agents, therapeutic vaccines are emerging as an effective means to activate the immune response by enhanced tumor antigen presentation and generating non-exhaustive cytotoxic T cells to improve anti-tumor immunity (140). These vaccines elicit the immune response by recognizing the specific epitopes expressed by tumor cells (140). Though there has been limited success with such vaccines so far, various clinical trials (clinicaltrials.gov) are now focusing on targeting tumor specific antigens (TSAs), which are exclusive to tumors and possess high immunogenicity (141). TSAs can be viral antigens or non-viral neoantigens generated by spontaneous somatic mutations in tumor microenvironment (142, 143). As many neoantigens are unique to either a small sub-population or specific to an individual patient, personalized cancer vaccines are gaining attention for precision targeting.

The most important factors to consider while designing a tailor-made cancer vaccine are: (a) Accurate identification of highly potent and immunogenic neoantigens capable of inducing a robust T cell immunity; (b) Calculative estimation of the probability of TSA-epitopes binding to MHC; (c) Neoantigen prioritization to predict the interaction of TCRs with MHC-neoepitope complex; (d) Selecting appropriate delivery platform for neoantigen based vaccine, which may include autologous dendritic cells (DCs), peptides, DNA, RNA, mRNA or viral vectors (144, 145). Autologous whole tumor cells mixed with immunomodulatory adjuvants, genetically modified autologous tumor cells, autologous cell derived exosomes, DC-tumor cell fusion vaccine, autologous DC-based or DNA/RNA/mRNA-based vaccines are a few examples of the ones undergoing clinical trials for personalized treatment (143, 146, 147). Recombinant viral vectors, such as Great Ape derived adenoviruses (GAd) and modified vaccinia virus Ankara (MVA) also serve as a great tool to trigger effective cytotoxic T cell response, using their intrinsic adjuvant properties (144). Anti-viral vector immunity can serve as a roadblock though, which can lead to ineffective immunity boost at re-administration. This challenge is being eradicated via a ‘heterologous-boost approach’ in various clinical studies, where involving different platforms can provide stronger immune response, examples include GAd - primed with MVA boost (148) or with self-amplifying RNA (149).

We are still in initial stages of personalized cancer vaccine development owing to the complexity and masking skills honed by tumor cells, which makes it difficult to recognize the distinguishing epitopes. A weakened immune system with immunosuppressive proteins expressed on tumor cells (such as PD-L1), loss of TSA expression or spontaneous alterations in antigen processing pathways, could be a few potential challenges (144, 150). Personalized vaccine manufacturing also involves a large cost, unique supply chain and the extensive process involved could cause a lag in timely treatment (144). As scientists are progressing this field forward, there is a need to further refine the cancer vaccine formulation and preparation workflows and make it more accessible to the wider group of patients in need.

## 5 Discussion

Diligent preclinical steps towards choosing the correct research platform (such as humanized mouse models (151, 152), organoids (153) or organ-on-chip (154)), appropriate drugs (155), and carefully curated experimentation strategies, serve as the foundation of any clinical trial. Undeniably, the molecular framework of tumors needs to be thoroughly studied at a deeper level to align with the required treatment regimens. Understanding resistance mechanisms and adopting alternative approaches is important from the early research steps (4, 156).

Combining these aspects with comprehensive AI-assisted technologies, such as NGS and multi-omics connects the pathway from preclinical (100, 157–160) to ‘personalized’ clinical stages (161). Since generative and multimodal AI models play a major role in patient diagnosis, trial design, planning, patient recruitment, drug delivery, digital monitoring, and data assessment, it is imperative to adopt a precautionary regulatory framework (162). European AI act and FDA have released regulatory policies for the use of AI in medical field (162, 163), however the rules need to be clearer and up to date as the field progresses. Keeping in mind the biases and limitations of large datasets generated from AI-based systems, the risk-benefit scale needs to be fine-tuned. Using real world evidence (RWE) also poses privacy and data confidentiality risks (164), which should be appropriately addressed. Furthermore, using high throughput screening methods for certain subpopulations would need comprehensive training models, absence of which may introduce bias or sub-par results (165). Thorough investigation of medical interventions is needed to be cautious of any false claims from personalized drug developers. Transparent evidence-based information sharing and finding accelerated solutions to unexpected contradictions is required to manage the fragmented regulation in clinical settings (162). Going hand in hand with the ethical considerations, the need for precision medicine outweighs any opposing schools of thought, recognizing that each life is important.

Significant progress has been seen in this field, with the launch of ‘Precision Medicine Initiative’ by Barack Obama in 2015 (166) and the Cancer Moonshot program (167), aiming to bring the public and private sectors together to provide a broader screening, diagnostic, therapeutic and supportive biomedical platform. Though many organizations are focused on developing cutting-edge technologies tailor-made to patients’ needs, we are still many steps away from accessible and affordable personalized healthcare for everyone in need. However, with precision oncology propelling cancer research, there is a gleam of hope for a healthier not-too-distant future.
